# Gap States Assisted MoO_3_ Nanobelt Photodetector with Wide Spectrum Response

**DOI:** 10.1038/srep04891

**Published:** 2014-05-08

**Authors:** Du Xiang, Cheng Han, Jialin Zhang, Wei Chen

**Affiliations:** 1 Department of Physics, National University of Singapore, 2 Science Drive 3, Singapore, 117542; 2 Department of Chemistry, National University of Singapore, 3 Science Drive 3, Singapore, 117543; 3 Graphene Research Centre, National University of Singapore, 3 Science Drive 3, Singapore, 117543; 4 National University of Singapore (Suzhou) Research Institute, 377 Lin Quan Street, Suzhou Industrial Park, Jiang Su, China, 215123

## Abstract

Molybdenum oxides have been widely investigated for their broad applications ranging from electronics to energy storage. Photodetectors based on molybdenum trioxide (MoO_3_), however, were seldom reported owing to their low conductivity and weak photoresponse. Herein we report a photodetector based on single MoO_3_ nanobelt with wide visible spectrum response by introducing substantial gap states via H_2_ annealing. The pristine MoO_3_ nanobelt possessed low electrical conductance and no photoresponse for nearly all visible lights. The H_2_ annealing can significantly improve the conductance of MoO_3_ nanobelt, and result in a good photodetector with wide visible spectrum response. Under illumination of 680 nm light, the photodetector exhibited high responsivity of ~56 A/W and external quantum efficiency of ~10200%. As corroborated by *in situ* ultraviolet photoelectron spectroscopy and X-ray photoelectron spectroscopy investigations, such strong wide spectrum photoresponse arises from the largely enriched gap states in the MoO_3_ nanobelt after H_2_ annealing.

Metal oxides nanomaterials have been widely used in optoelectronic nanodevices^1^, solar cells^2^ and photocatalysis^3^. However, their wide bandgap limits the applications in ultraviolet region^4^,^5^,^6^. It has been demonstrated that generating mid-gap states in these wide bandgap semiconductors can extend the photoactive region to visible or even infrared range, and hence significantly improve the efficiency of the optoelectronic devices and photocatalysts^7^,^8^,^9^,^10^. In order to produce substantial gap states in these wide bandgap semiconductors, various approaches have been proposed, including intercalating metal or nonmetal dopants in the wide bandgap semiconductors to introduce donor or acceptor states in various positions above the valance band and altering the degree of doping to modify the gap states^11^,^12^,^13^,^14^,^15^,^16^. For metal oxides, one effective way to generate gap states is to remove oxygen ions in the lattice, and hence the formation of oxygen vacancies. These oxygen vacancies are vitally important to determine the electronic and optical properties of metal oxides^17^,^18^,^19^. Annealing the nanostructures of metal oxides in reducing gas is effective to obtain such oxygen vacancies^19^,^20^. Wang et al. demonstrated that oxygen vacancies were generated in rutile TiO_2_ nanowire arrays by annealing the samples in H_2_ atmosphere. These oxygen vacancies served as donor states to strongly improve the light absorption^9^. Davazoglou et al. succeeded to utilize oxygen vacancies in WO_3_ and MoO_3_ films based organic light-emitting diodes and solar cells to improve their performance^17^,^18^. Recently, it was found that introducing large amounts of lattice disorder in nanophase TiO_2_ can generate substantial gap states, and hence extend the light absorption edge to ~1200 nm, thereby leading to the remarkably enhanced photocatalytic efficiency^21^,^22^.

Attributed to the reduced dimensionality and large surface-to-volume ratio, photodetectors based on one dimensional (1D) nanomaterials possess two major advantages compared to their bulk counterparts, including high sensitivity and high quantum efficiency^23^,^24^,^25^. However, the photodetectors based on 1D nanomaterials with large bandgap only works under the light with narrow spectra range^5^. Introducing considerable gap states in such wide bandgap 1D nanomaterials can help broadening their photoresponse spectra region.

The molybdenum trioxide (MoO_3_), as an intrinsic n-type II-VI semiconductor with wide bandgap (~3.2 eV), has been extensively utilized in organic electronics as efficient anode interfacial layers owing to its high work function^26^. Moreover, the MoO_3_ nanostructures have also been heavily investigated as effective photocatalyst in pollution degradation^27^,^28^. However, due to their low intrinsic conductivity and weak photoresponse^29^, MoO_3_ based optoelectronic nanodevices are rarely reported. In this paper, a photodetector with wide visible spectrum response based on single MoO_3_ nanobelt treated by annealing in H_2_ was proposed and carefully examined. The intrinsic MoO_3_ nanobelt device exhibited low electrical conductance and no photoresponse for the visible spectrum. After H_2_ annealing, the conductance of MoO_3_ nanobelt was largely enhanced; at the same time, the photodetector possessed wide visible spectrum response. The responsivity and external quantum efficiency of the photodetector under the illumination of 680 nm light can reach as high as 56 A/W and 10200%, respectively. *In situ* ultraviolet photoelectron spectroscopy (UPS) and X-ray photoelectron spectroscopy (XPS) measurements indicate the significant enrichment of gap states in MoO_3_ after H_2_ annealing, thereby leading to the excellent photoresponse in the wide visible spectra region.

## Results

**Figure 1:**
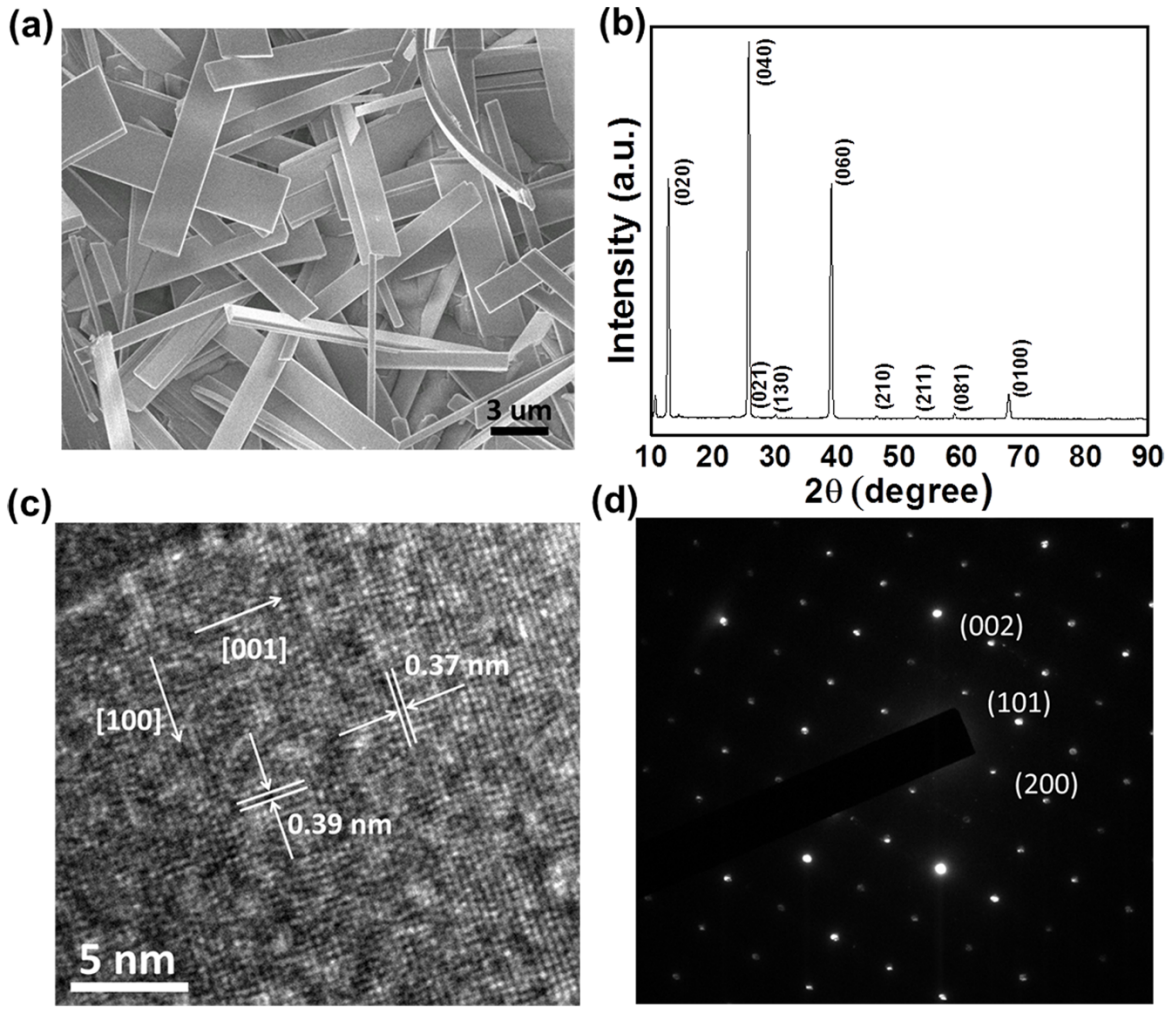
Morphology and lattice structure of the as-grown MoO_3_ nanobelts. (a) SEM image and (b) XRD pattern of the as-grown MoO_3_ nanobelts. (c) HRTEM image of a single nanobelt, and (d) the corresponding SAED pattern.

Figure 1a displays a typical SEM image of as-grown MoO_3_ nanobelts. The sample showed the widths ranging from 1 to 4 um and lengths from 10 to 25 um. The average thickness of nanobelts was about 100 nm. The XRD pattern (shown in figure 1b) is in good agreement with the orthorhombic structure of MoO_3_ phase, with lattice constants of a = 3.96 Å, b = 13.86 Å, and c = 3.7 Å (JCPDS 05-0508). The high-resolution transmission electron microscopy (HRTEM) and selected area electron diffraction (SAED) images of individual nanobelt are shown in figure 1c and 1d, respectively. The TEM information revealed that the nanobelt was single crystalline with longitudinal direction preferentially along the <001> direction.

**Figure 2:**
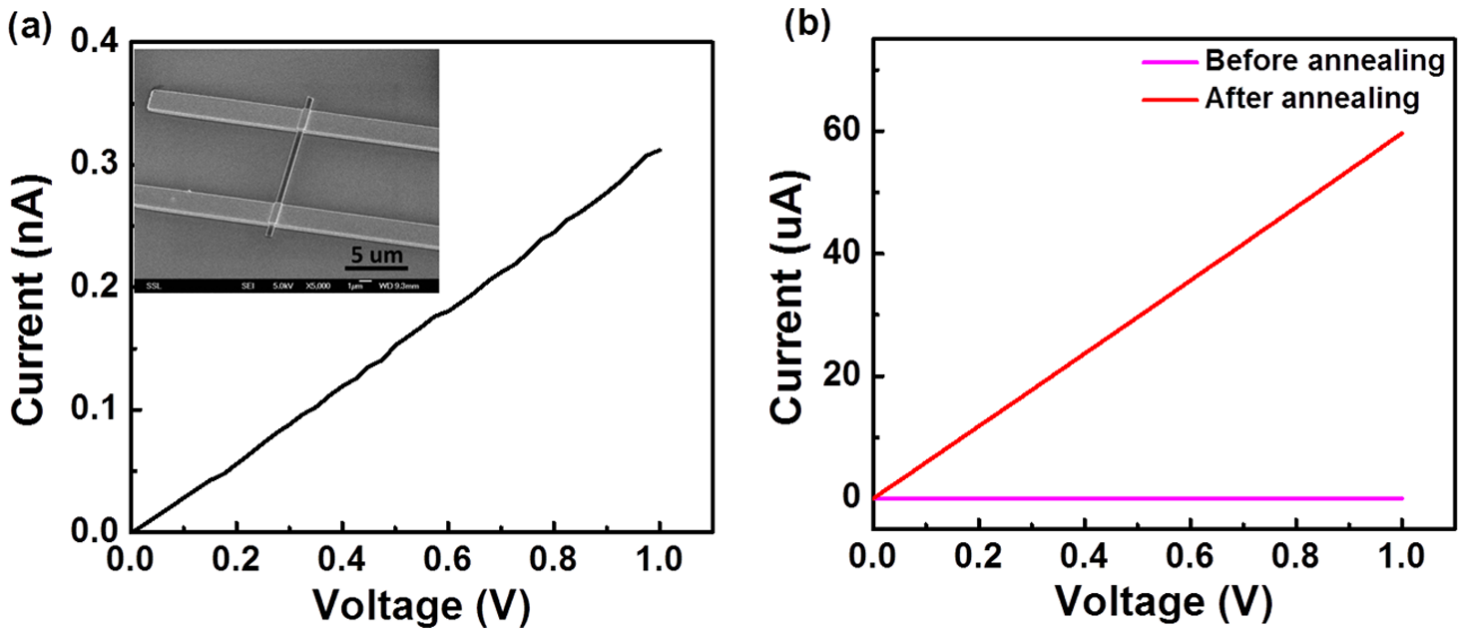
Electronic measurements of MoO_3_ nanobelt device before and after H_2_ annealing. (a) *I–V* curve of the single MoO_3_ nanobelt before annealing. The inset shows a SEM image of the single nanobelt device. (b) Comparison of the *I–V* curves before and after H_2_ annealing.

In order to explore the electrical transport properties of as-grown MoO_3_ nanobelts, the single nanobelt was configured with two Cr/Au (50 nm/100 nm) contacts via the conventional e-beam lithography (EBL) process. The inset of Figure 2a shows the SEM image of a typical fabricated device with the conduction channel length of 6 μm. The typical current-voltage (*I–V*) characteristic of the fabricated device is illustrated in Figure 2a. The good linearity of *I–V* curve reveals the ohmic contact between electrodes and MoO_3_ nanobelt. The conductance was calculated to be ~3.14 × 10^−10^ S. Through the annealing treatment in H_2_ atmosphere, the conductance of the MoO_3_ nanobelt device dramatically increased to ~5.96 × 10^−5^ S by 5 orders of magnitude, as demonstrated in Figure 2b.

**Figure 3:**
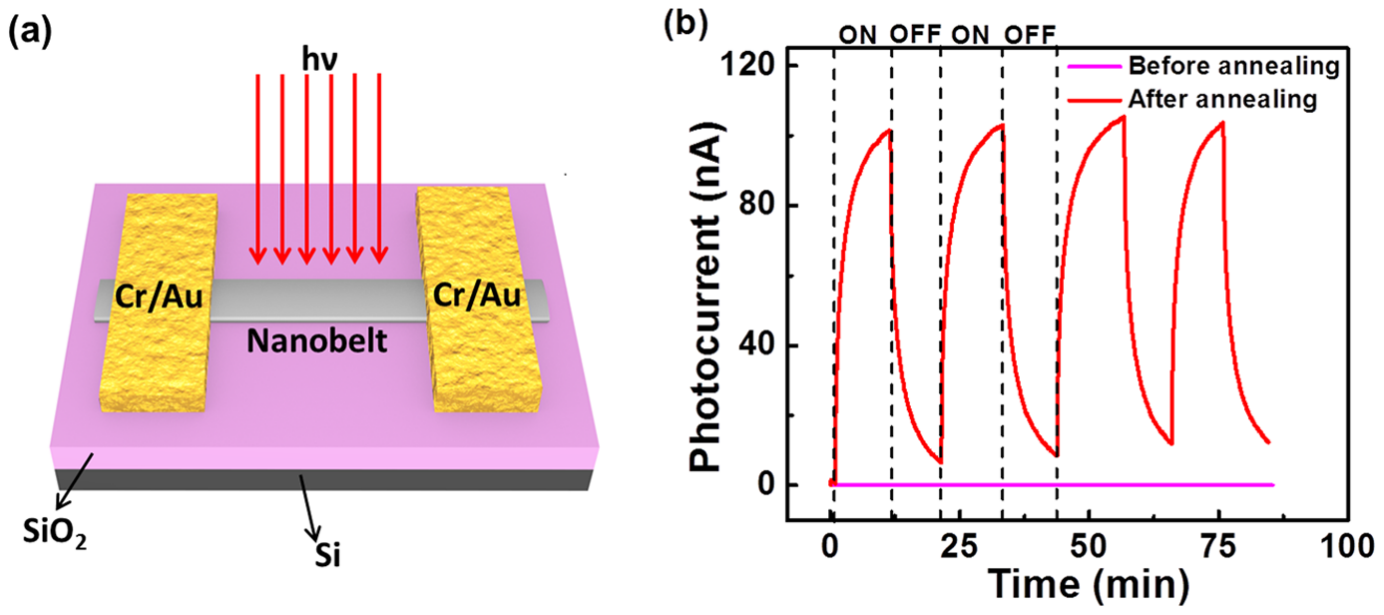
Photodetector based on single MoO_3_ nanobelt. (a) Schematic illustration of MoO_3_ nanobelt device for photocurrent measurement. (b) Time dependent photoresponse of MoO_3_ device before and after annealing under 660 nm laser illumination at 0.1 V bias voltage.

The schematic diagram of single MoO_3_ nanobelt based photodetector is exhibited in Figure 3a. Figure 3b shows the time dependent photocurrent measurements of MoO_3_ nanobelt before and after H_2_ annealing via alternately switching on and off a 660 nm laser with the power density of 112.3 mW/cm^2^. The photocurrent (*ΔI*) is defined as: *ΔI = I_p_* – *I_d_*, where *I_d_*, *I_p_* represents the current at bias voltage of 0.1 V in the dark and under light illumination, respectively. Prior to annealing process, the pristine MoO_3_ nanobelt exhibited nearly no photoresponse illuminated by the incident light. In contrast, the significant photoresponse was detected for the H_2_ annealed nanobelt with the excellent reproducibility and apparent photocurrent as high as ~100 nA. This remarkable photoresponse of the annealed device was also observed upon the illumination over a wide visible spectrum; while the MoO_3_ nanobelt before annealing demonstrated almost zero photocurrent under these visible lights with different wavelength.

**Figure 4:**
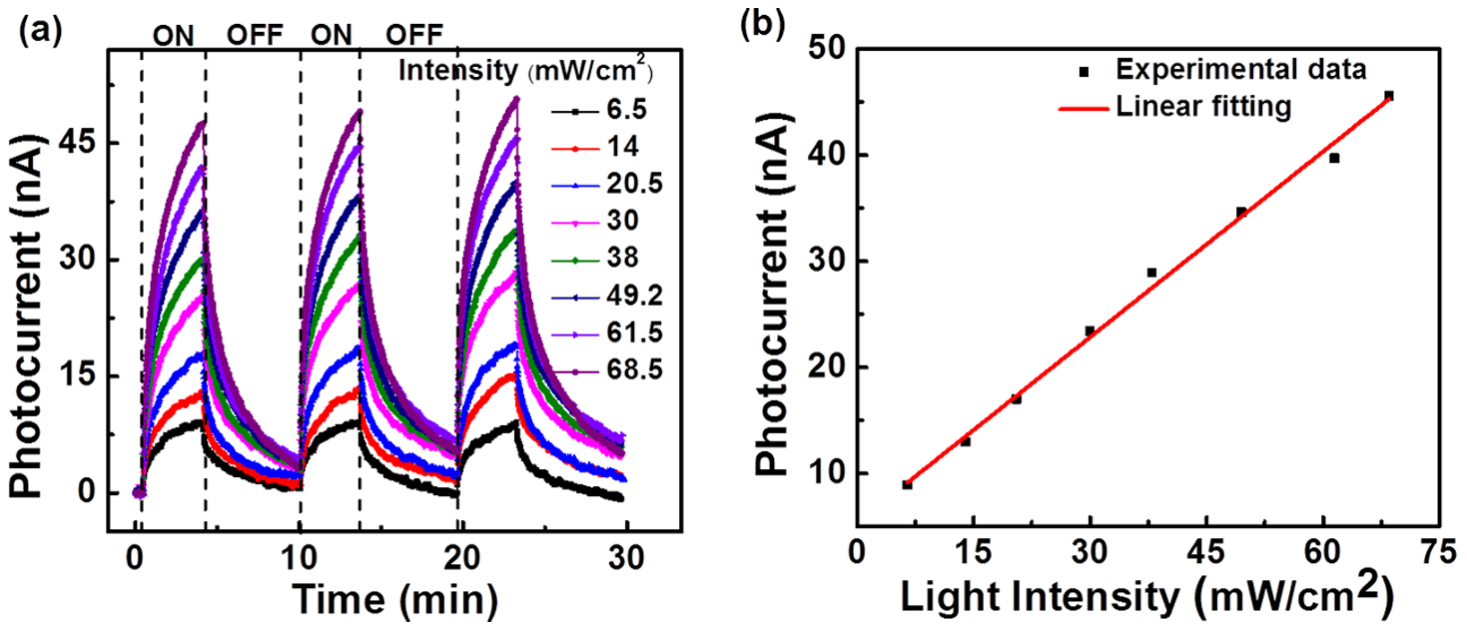
Photocurrent versus light intensity of annealed MoO_3_ nanobelt. (a) Time dependent photoresponse of MoO_3_ nanobelt device after annealing under 532 nm laser with varying intensities at bias voltage of 0.1 V. (b) Plot of the photocurrent as a function of laser intensity.

The relationship between photocurrent and intensity of incident light for H_2_ annealed MoO_3_ nanobelt was also examined. Figure 4a exhibits the real-time photoresponse of annealed nanobelt irradiated by a 560 nm laser with varying intensities. The photocurrent increased from 8.9 nA to 45.6 nA by increasing the laser intensity from 6.5 mW/cm^2^ to 68.5 mW/cm^2^. The corresponding photocurrent versus light intensity plot is shown in Figure 4b. It indicates that the photocurrent increases almost linearly as a function of the intensity of incident light. It is believed that the density of photo-induced charge carrier and hence the photocurrent linearly depends on the absorbed photo flux^23^, in good agreement with our experimental results. Such linear dependence of photocurrent as a function of light intensity reveals the potential application of the annealed MoO_3_ nanobelt as light power detectors.

**Figure 5:**
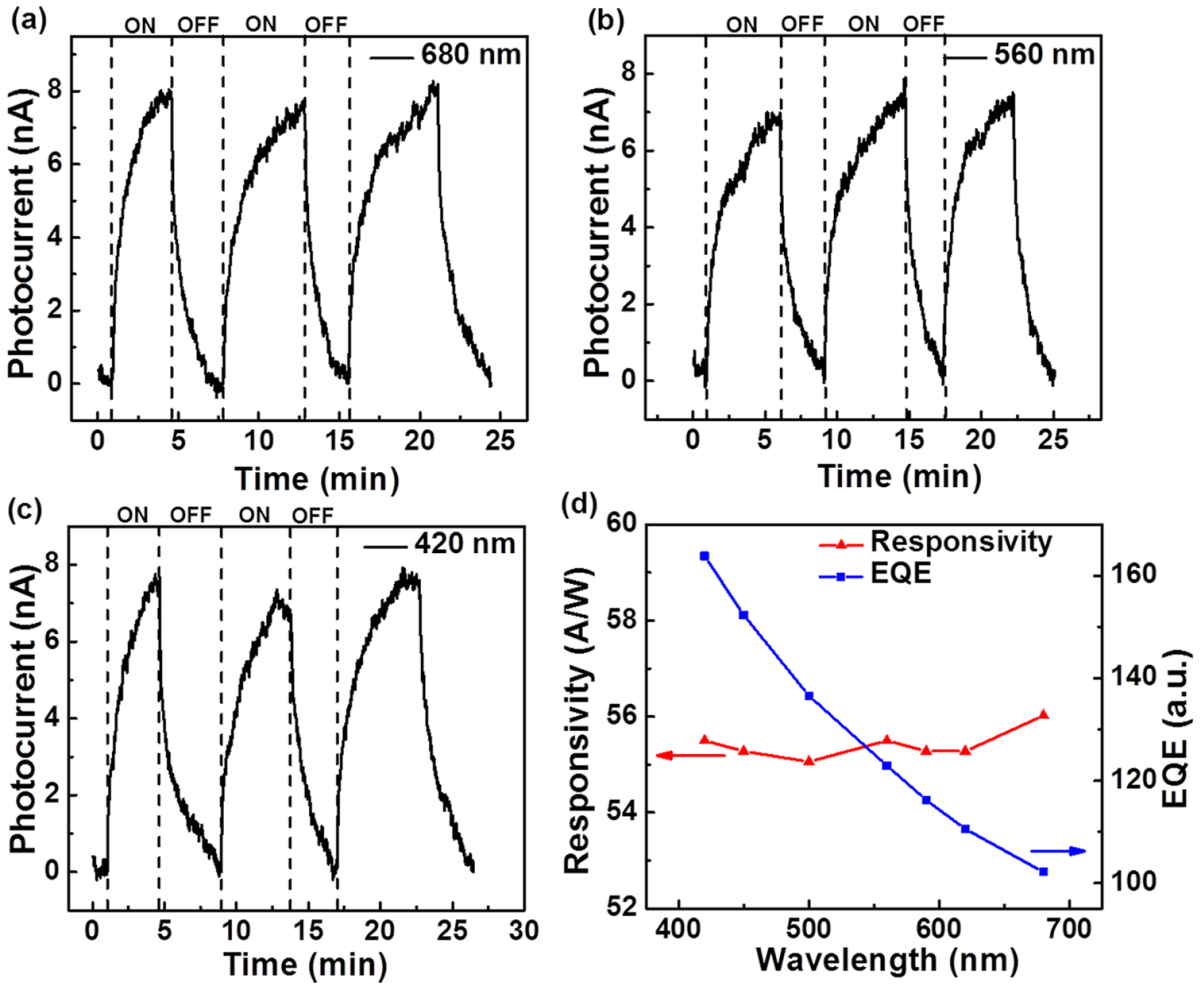
Wavelength dependence of annealed MoO_3_ nanobelt photodetector. Photoresponse of annealed MoO_3_ nanobelt device under the light with different wavelength: (a) 680 nm (b) 560 nm (c) 420 nm. The intensity of light is kept the same at 5.6 mW/cm^2^. (d) Plot of the responsivity and EQE versus light wavelength.

In order to probe the wavelength dependence of the photosensitivity of MoO_3_ nanobelt photodetector, the time dependent photoresponse of the annealed device was measured under the exposure to visible lights of selected wavelengths ranging from 400 nm to 700 nm with the same intensity of 5.6 mW/cm^2^ (as shown in Figure 5a–5c). The significant photocurrent under these visible lights with different wavelength indicates the wide spectrum response of the annealed nanobelt photodetector. Moreover, nearly reserved photocurrent through the visible spectrum was observed, suggesting the uniform visible light photoresponse for the annealed MoO_3_ nanobelt.

The spectra responsivity (*R_λ_*) and external quantum efficiency (EQE) are two critical parameters to evaluate the quality of photodetectors, where *R_λ_* is defined as the photocurrent generated per unit power of incident light on the effective area of a photodetector, and EQE is the number of electrons detected per incident photon. The large values of *R_λ_* and EQE suggest high sensitivity for photodetectors. *R_λ_* and EQE can be expressed as^30^: 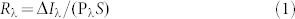

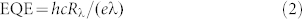
where *ΔI*_λ_ is the photocurrent induced by the incident light of wavelength λ, P_λ_ is the light intensity, *S* is the effective illuminated area, and *h, c, e* represent the Plank constant, velocity of light, and charge of electron, respectively. At the bias voltage of 0.1 V, the *R_λ_* of H_2_ annealed MoO_3_ nanobelt for the selected visible wavelengths was calculated to be in the range of 55 to 56 A/W (shown in figure 5d). This is much higher than many reported photodetectors based on both 1D and two dimensional (2D) materials, such as ZnS nanobelts (~0.12 A/W)^31^, SbSe_3_ nanowires (~8.0 A/W)^32^, ZnSe nanobelts (~20 A/W)^33^ and 2D materials, such as graphene (~1 mA/W)^34^, single layer MoS_2_ (~7.5 mA/W)^35^, multi-layer GaS nanosheets (~4.2 A/W)^36^, but still significantly lower than that of In_2_Se_3_ nanowires (~89 A/W)^37^, ZnTe nanowires (~360 A/W)^38^, and CdSe nanobelts (~1400 A/W)^39^ based photodetectors. Moreover, the EQE of annealed device can be determined as high as 16300% for the wavelength of 420 nm, and gradually decreased to 10200% as the wavelength of incident light increased to 680 nm, revealing superior device performance of the MoO_3_ nanobelt based photodetector.

## Discussion

**Figure 6:**
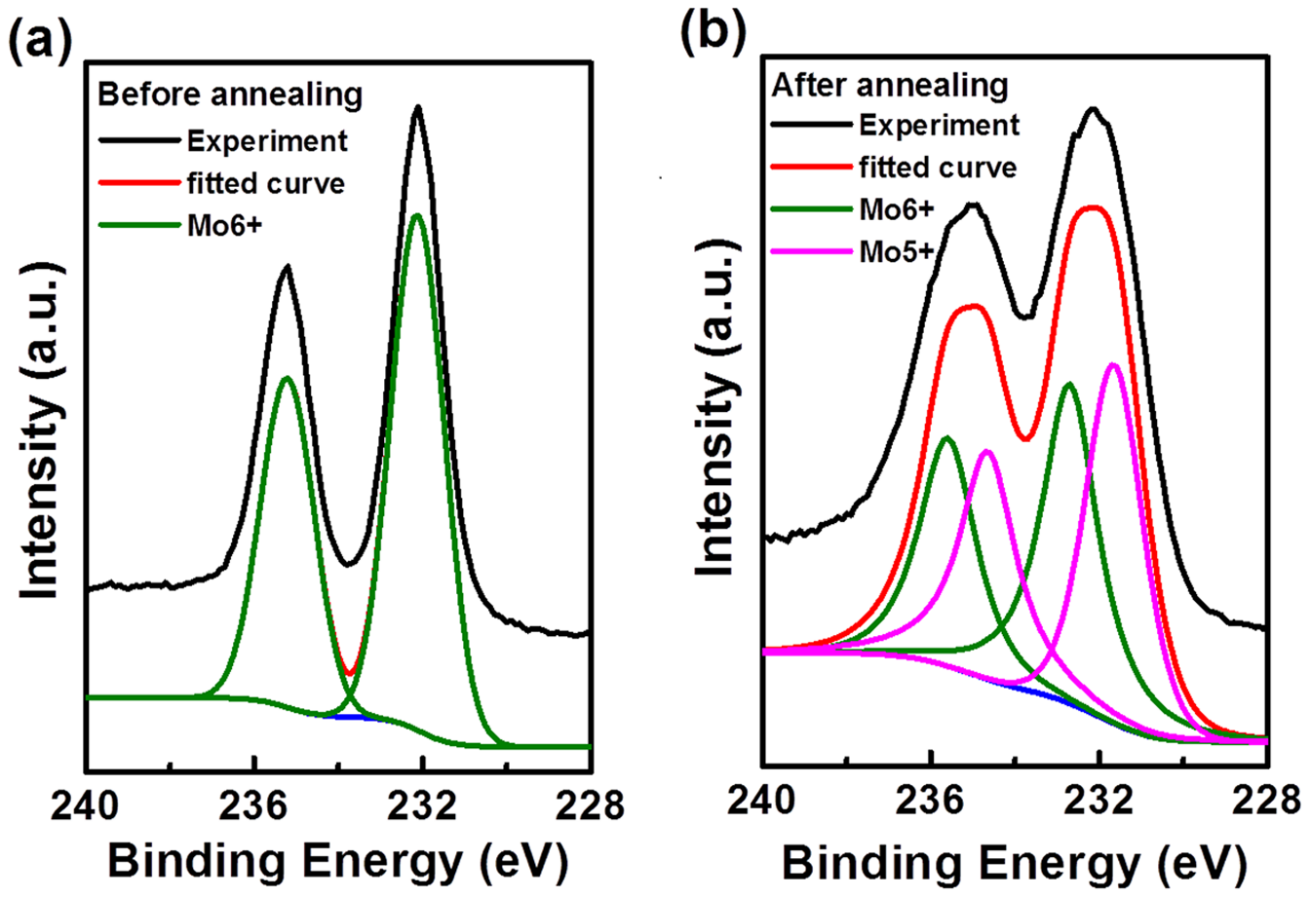
XPS investigation. XPS spectra of MoO_3_ film for Mo 3d core level (a) before and (b) after annealing. The experiment data are fitted with the Gaussian/Lorentzian mixed functions.

To further investigate the mechanism of strong photoresponse to wide visible spectrum for H_2_ annealed MoO_3_ nanobelt photodetector, XRD and *in situ* XPS/UPS measurements were conducted on as-grown MoO_3_ nanobelts and thermally deposited MoO_3_ thin film before and after the H_2_ annealing process, respectively. As shown in Figure S1, H_2_ annealing did not induce any crystal structure change of the MoO_3_ nanobelts. Figure 6a and 6b show the Mo 3d core level XPS spectra of the *in situ* grown MoO_3_ film (10 nm) before and after H_2_ annealing, respectively. These two core level spectra were fitted with Gaussian/Lorentzian mixed functions. In Figure 6a, the Mo 3d_5/2_ and 3d_3/2_ peaks located at the binding energy of 232.11 eV and 235.21 eV can be assigned to the 6+ oxidation state of MoO_3_ phase, in accordance with the previous reports^40^. This suggests that Mo^6+^ dominates the MoO_3_ layer before the H_2_ annealing. After H_2_ annealing, the Mo 3d peaks were apparently broadened arising from the appearance of Mo^5+^ oxidation state (as shown in Figure 6b). This reveals that large quantity of oxygen vacancies were introduced in MoO_3_ through H_2_ annealing, reducing the Mo atoms neighboring to the oxygen vacancies from the 6+ state to the 5+ state. The corresponding O 1s XPS spectra shown in Figure S2 also indicate the significant enhancement of oxygen vacancies after annealing, in good agreement with Mo 3d XPS spectra. The H_2_ annealing can also significantly increase the charge carrier (electron) concentration and induce obvious n-type doping of MoO_3_, thereby significantly enhancing the conductivity of MoO_3_ nanobelts.

**Figure 7:**
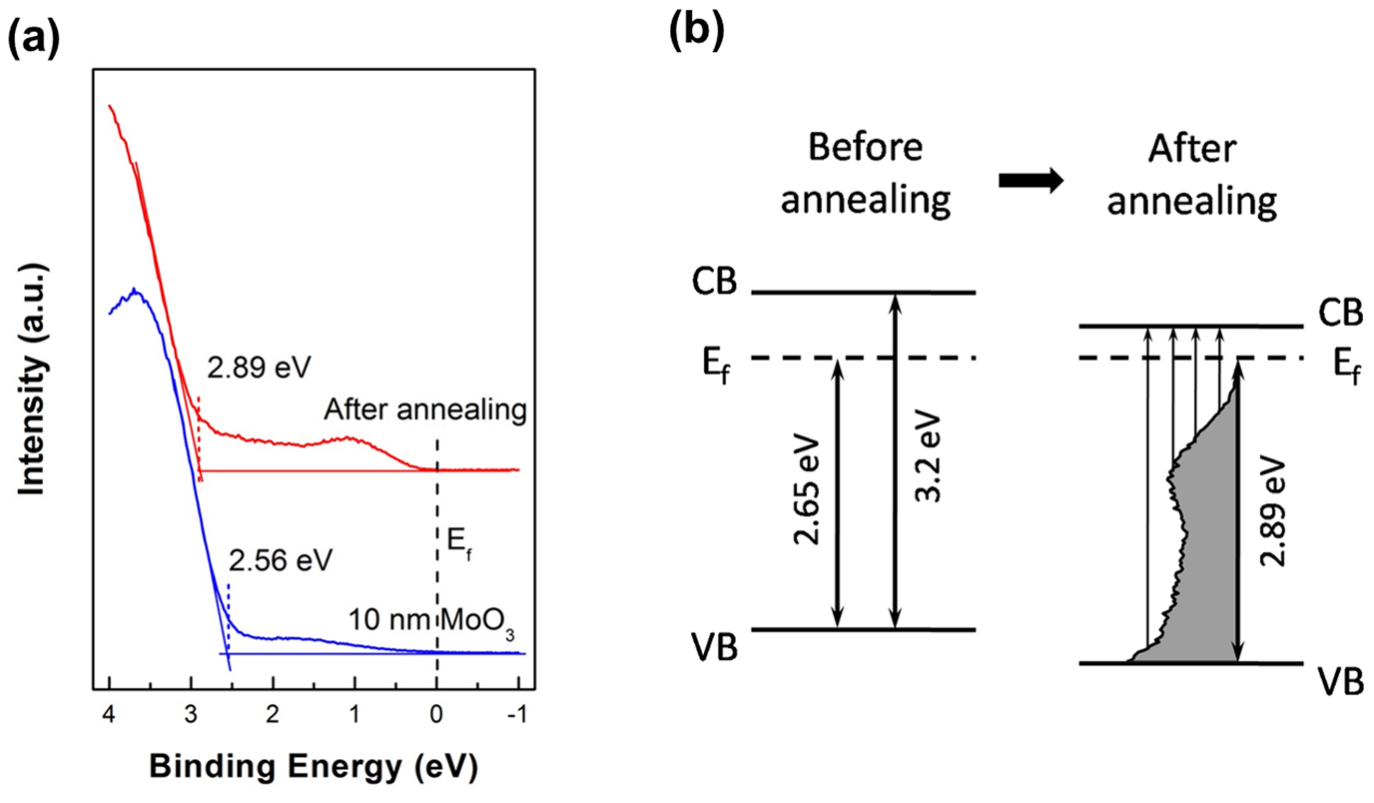
UPS characterization. (a) UPS spectra of the low binding energy region near the Feimi level for MoO_3_ film before and after annealing. (b) Schematic diagram of the energy level alignment for MoO_3_ film before and after annealing.

H_2_ annealing of MoO_3_ can induce the formation of substantial gap states to facilitate the aforementioned wide-range visible light response in MoO_3_ nanobelt based photodetectors. This hypothesis can be corroborated by *in-situ* UPS measurements. Figure 7a shows the UPS spectra of MoO_3_ thin film before and after H_2_ annealing at the low binding energy region near the Fermi level. By linear extrapolation of the low binding energy onset, the valence band edge of MoO_3_ layer without annealing was measured to be ~2.56 eV. After H_2_ annealing, the valence band edge was located at ~2.89 eV below the Fermi level. This indicates that the Femi level moved 0.33 eV closer to the conduction band and hence a more significant n-type doping of MoO_3_, in agreement with the XPS results. After H_2_ annealing, the intensity of the gap states located between the Femi level and the valence band edge was significantly enhanced. Moreover, these gap states substantially extended towards the Fermi level. As shown by the energy level diagram of MoO_3_ before and after annealing in Figure 7b, upon light illumination, such annealing process induced gap states offer many possible routes for electrons to be excited from gap states to the conduction band. This can significantly improve the photoresponse under the illumination of visible lights with different wavelength, making H_2_ annealed MoO_3_ nanobelt as an effective photodetector with wide spectrum response.

In conclusion, we report a MoO_3_ nanobelt based photodetector with wide spectrum response in the visible light region assisted through the H_2_ annealing induced gap states with high density. The as-grown MoO_3_ nanobelt exhibited low conductance and nearly no photoresponse under visible light irradiation. After H_2_ annealing, the conductance of MoO_3_ nanobelt was dramatically enhanced; moreover, the photodetector possessed wide visible spectrum photoresponse with high responsivity and EQE. As corroborated by *in situ* XPS and UPS measurements, such excellent photodetector with wide spectrum response mainly resulted from the significantly enriched gap states in H_2_ annealed MoO_3_ nanobelt. This work demonstrates the possibility to extend the wide bandgap metal oxide nanomaterials based optoelectronics devices or photocatalysts with efficient visible light response through the introduction of the high intensity of carefully engineered gap states.

## Methods

### Material Preparation and Characterization

The MoO_3_ nanobelts were synthesized by adopting the previously reported method^41^. A molybdenum foil (size of 10 mm × 10 mm × 0.05 mm, 99.9% Mo) was used as the Mo source to grow MoO_3_ nanobelts. Firstly, the molybdenum foil was polished to remove the oxide layer and washed in acetone and distilled water via sonication. It was then placed on a ceramic digital stirring hotplate with a glass slide (35 mm × 50 mm × 150 um in size) covering on it. The hotplate was heated at 480°C for 2 days in the air ambient. After heating, the hotplate was allowed to cool down to room temperature. MoO_3_ nanobelts were grown on the glass slide. The nanostructures were characterized by scanning electron microscope (JEOL JSM-6400F), X-ray diffraction (Philip PW 127), and transmission electron microscope (JEOL TEM 2010F).

### Device Fabrication and Characterization

Single MoO_3_ nanobelt based device was fabricated by the standard lithography procedures. The as-grown MoO_3_ nanobelts on glass were dispersed in ethanol by sonication. The nanobelts suspension was subsequently dropped on the heavily p-doped Si substrate (resistivity < 0.005 Ω·cm) with 300 nm thermal oxide followed by drying under nitrogen. Two electrodes with bonding pads were precisely pattered on the single nanobelt using the conventional e-beam lithography (EBL) technique, followed by thermal deposition of Cr (50 nm) and Au (100 nm) bilayer as the metal contact. After lift-off process, the fabricated devices were wire-bonded on a LCC chip carrier for electrical measurements. The annealing process of as-made MoO_3_ nanobelt devices was conducted in H_2_/Ar (10%) at 300°C for 1 hour. All the electrical and optoelectronic measurements were carried out in high vacuum (~10^−8^ mbar) using an Agilent B2912A source measurement unit. The light sources utilized in our experiments contain 660 nm laser, 532 nm laser, and 500 W xenon light source configured with a monochromator to give a continuous spectrum output. The power of the incident light was calibrated by THORLABS GmbH (PM 100A) power meter.

### XPS and UPS Measurements

MoO_3_ thin film was grown on the Si (111) substrate coated with native oxide layer (1–10 Ω·cm) via thermal evaporation in an ultra-high-vacuum (UHV) chamber with a base pressure of ~2 × 10^−9^ mbar. The highly purified MoO_3_ source was thermally evaporated onto Si substrate from a Knudsen cell (Creaphys, Germany) at the temperature of 490°C. The thickness of the grown MoO_3_ layer was estimated by the attenuation of Si 2p peak and further calibrated by a quartz crystal microbalance (QCM). *In situ* XPS and UPS measurements were carried out in an analysis chamber of base pressure ~1 × 10^−10^ mbar with Al Kα (1486.6 eV) and He I (21.2 eV) as the excitation source. The as-grown MoO_3_ thin film was *in situ* annealed in H_2_ atmosphere at 300°C under the pressure of 5 × 10^−5^ mbar for 1 hour.

## Author Contributions

D.X. and C.H. contributed equally to this paper. D.X. and W.C. designed the experiments. D.X., C.H. and J.Z. performed the experiments. D.X., C.H. and W.C. wrote the main manuscript text. D.X., C.H. and J.Z. prepared figures 1-7. D.X. prepared figures S1 and S2. All authors reviewed the manuscript.

## Supplementary Material

Supplementary Information Supporting materials
