# Supplementary material for: Gap States Assisted MoO3 Nanobelt Photodetector with Wide Spectrum Response
Source: Sci Rep. 2014 May 8;4:4891. doi: 10.1038/srep04891 (PMC4013929; doi:10.1038/srep04891)
Supplement: Supplementary Information — Supporting materials [file srep04891-s1.pdf]

Supporting Materials

Gap States Assisted MoO_3 Nanobelt Photodetector with Wide Spectrum Response

Du Xiang[1], Cheng Han[1], Jialin Zhang[1] & Wei Chen[1,2,3,4*]

[1]Department of Physics, National University of Singapore, 2 Science Drive 3, Singapore, 117542

[2]Department of Chemistry, National University of Singapore, 3 Science Drive 3, Singapore, 117543

[3]Graphene Research Centre, National University of Singapore, 3 Science Drive 3, Singapore, 117543

[4]National University of Singapore (Suzhou) Research Institute, 377 Lin Quan Street, Suzhou Industrial Park, Jiang Su, China, 215123

* To whom correspondence should be addressed: W.C (phycw@nus.edu.sg)

1. XRD patterns for annealed MoO_3 nanobelts

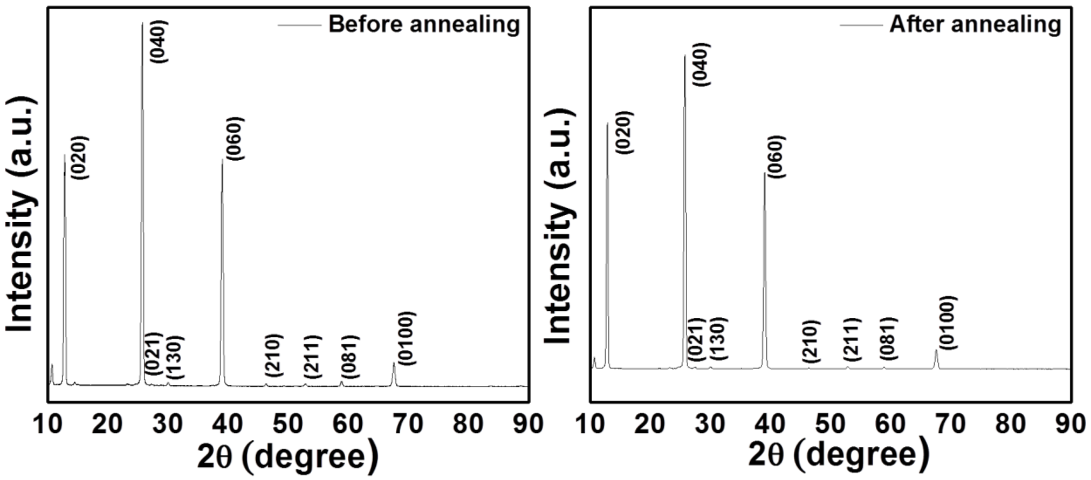

Figure S1. XRD patterns for as-grown MoO_3 nanobelts before (left) and after (right) H_2 annealing.

2. **XPS investigation for O 1s**

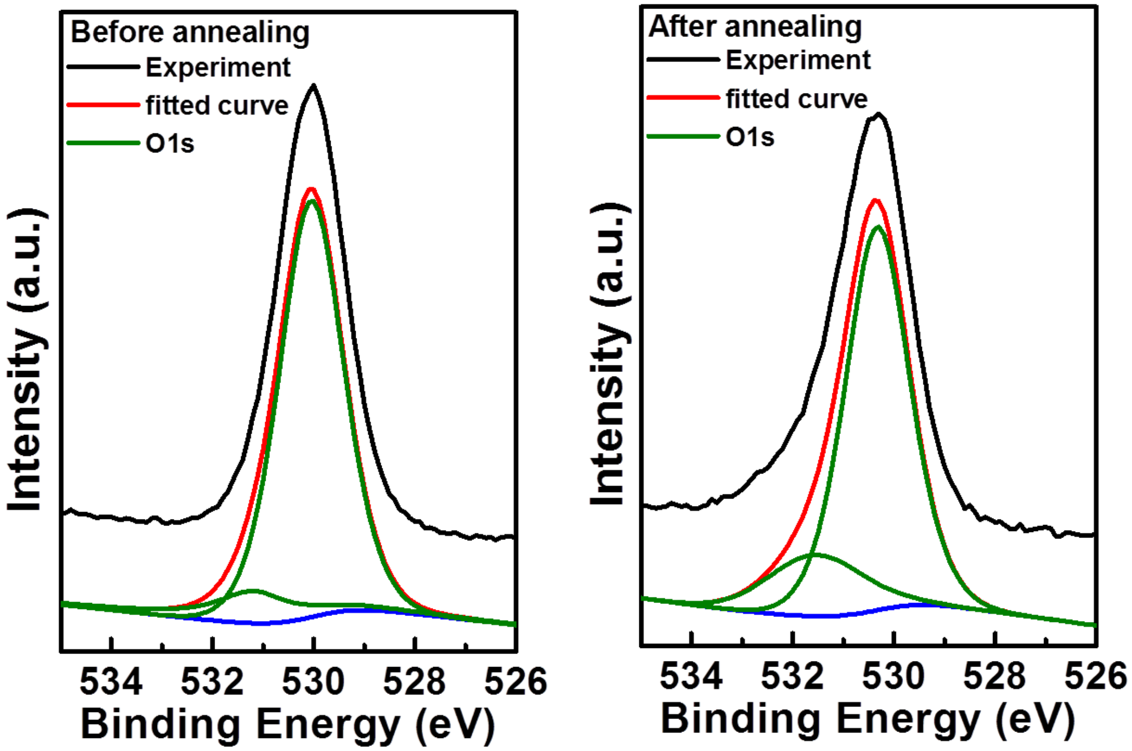

Figure S2. XPS spectra of MoO_3 film for O 1s core level (left) before and (right) after annealing. The experiment data are fitted with the Gaussian/Lorentzian mixed functions.

Figure S2 shows the O 1s XPS spectra of MoO_3 thin film before and after H_2 annealing. The O 1s peaks can be fitted into two peaks. The peak with lower binding energy (530 eV) corresponds to O^{2-} in normal MoO_3 matrix. The second peak at higher binding energy position (531.2 eV) originates from the oxygen-deficient components.[1-5] It was observed that the relative intensity of the higher binding energy O 1s peak increased largely after annealing, indicating the significant enrichment of oxygen vacancies, which is consistent with the our Mo 3d XPS spectra in Figure 6.

References

1. Chen, Y. et al. Single-crystalline orthorhombic molybdenum oxide nanobelts: synthesis and photocatalytic properties. *CrystEngComm* **12**, 3740-3747 (2010).
2. Sun, Y. et al. Inverted Polymer Solar Cells Integrated with a Low-Temperature-Annealed Sol-Gel-Derived ZnO Film as an Electron Transport Layer. *Adv. Mater.* **23**, 1679-1683 (2011).
3. Wei, X. Q. et al. Blue luminescent centers and microstructural evaluation by XPS and Raman in ZnO thin films annealed in vacuum, N_2 and O_2. *Physica B* **388**, 145-152 (2007).
4. Lee, J., Chung, J., Lim, S. Improvement of optical properties of post-annealed ZnO nanorods. *Physica E* **42**, 2143-2146 (2010).
5. Wang, Z. G., Zu, X. T., Zhu, S., Wang, L. M. Green luminescence originates from surface defects in ZnO nanoparticles. *Physica E* **35**, 199-202 (2006).
